# Costing Analysis of Scalable Carbon‐Based Perovskite Modules Using Bottom Up Technique

**DOI:** 10.1002/gch2.202100070

**Published:** 2021-10-31

**Authors:** Priyanka Kajal, Bhupesh Verma, Satya Gangadhara Rao Vadaga, Satvasheel Powar

**Affiliations:** ^1^ School of Engineering Indian Institute of Technology Mandi Mandi Himachal Pradesh 175005 India; ^2^ Center for Study of Science Technology and Policy Bangalore Karnataka 560094 India; ^3^ Ananyavijaya Consultancy LLP Bangalore Karnataka 562107 India; ^4^ School of Technology and Business Studies Energy Technology Högskolan Dalarna Falun 79188 Sweden

## Abstract

In recent years, perovskite solar cells (PSCs) have achieved a remarkable power conversion efficiency of 25.5%, indicating that they are a promising alternative to dominant Si photovoltaic (PV) technology. This technology is expected to solve the world's energy demand with minimal investment and very low CO_2_ emissions. The market has shown a lot of interest in PSCs technology. A technoeconomic analysis is a useful tool for tracking manufacturing costs and forecasting whether technology will eventually achieve market‐driven prices. A technoeconomic analysis of a 100 MW carbon‐based perovskite solar module (CPSM) factory located in India is presented in this paper. Two CPSMs architectures—high‐temperature processed CPSMs (Module A) and low‐temperature processed CPSM's (Module B)—are expected to offer minimum sustainable prices (MSPs) of $ 0.21 W^‐1^ and $ 0.15 W^‐1^. On the basis of MSP, the levelized cost of energy (LCOE) is calculated to be 3.40 ¢ kWh^‐1^ for module A and 3.02 ¢ kWh^‐1^ for module B, with a 10‐year module lifetime assumption. The same modules with a 25‐year lifespan have LCOEs of 1.66 and 1.47 ¢ kWh^‐1^, respectively. These estimates are comparable to market dominant crystalline silicon solar modules, and they are also favorable for utilizing perovskite solar cell technology.

## Graphical abstract

A technoeconomic analysis of two widely reported carbon based perovskite solar cells architectures i.e. the triple mesoscopic (high *T*) and low temperature (*T*) architectures is presented. Minimum sustainable price (MSP), sensitivity analysis, and levelized cost of energy (LCOE) is calculated for these architectures. The calculated MSPs for High *T* and Low *T* modules are 21.06 ¢ W^‐1^ and 15.48 ¢ W^‐1^, respectively.

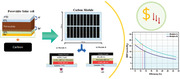

## 1 Introduction

Carbon dioxide emissions are a global issue that are causing the earth's temperature to rise and, as a result, affecting people's livelihoods. According to IRENA's annual energy‐related CO_2_ emission calculation for 2019, the earth's CO_2_ emissions will be 33 Gt by 2050. Three potential solutions include electrification of transportation, massive amounts of renewable energy installations, and increased energy efficiency. As a result, CO_2_ emissions can be reduced by 70%, resulting in 9.8 Gt in 2050.^[^ ^1^ ^]^ Among these, increased renewable energy production is thought to play a significant role in CO_2_ reduction. Taking this into consideration, all countries began investing in the production and installation of renewable energy sources such as solar, wind, biomass, and hydropower, among others.^[^ ^2^ ^]^ Among the various renewable energy sources, solar is seen as the most promising and widely available. As a result, significant research is being conducted to improve the performance of solar PV. Silicon (Si) photovoltaics (PV) is the dominant solar PV technology, with a good power conversion efficiency (PCE) and stability.^[^ ^3^ ^]^ However, the production of Si PV manufacturing is a time‐consuming process and with a production of 40 g CO_2_ eq kWh^−1^.^[^ ^4^, ^5^ ^]^ Given that this research is aimed at identifying alternative PV materials with lower material utilization, lower cost, and ease of processing. Perovskite PV has achieved a remarkable PCE of 25.6% in a few years of research with easy processability and negligible CO_2_ emissions.^[^ ^6^ ^]^ As a result, it is assumed to be a Si PV technology alternative. Several solar cell manufacturing companies, including Oxford PV,^[^ ^7^ ^]^ Solliance,^[^ ^8^ ^]^ wonder solar,^[^ ^9^ ^]^ Solaronix,^[^ ^10^ ^]^ Saule technologies,^[^ ^11^ ^]^ have emerged in recent years, indicating that perovskite PV is a promising future PV technology.

Considering the need to transition to renewable technology and contribute to the global CO_2_ emission reduction target, the Indian government also announced 100 and 300 Giga Watt (GW) solar photovoltaic (PV) installation targets for 2022 and 2030, respectively.^[^ ^12^, ^13^ ^]^ India currently has approximately 35 GW of installed PV capacity, the vast majority of which is based on crystalline‐silicon (c‐Si) technology.^[^ ^14^ ^]^ India's solar photovoltaics production capacity is 3.2 GW cell manufacturing and 8.4 GW module manufacturing.^[^ ^15^ ^]^ Polysilicon, ingot, and wafer are upstream supply chain components that are not manufactured in India at a large scale. India imports a massive portion of wafers, cells, and modules consumed in India. The COVID‐19 pandemic has caused supply chain disruptions in recent years, resulting in a significant drop in Indian solar PV installations. The situation does not seem to change any time soon, so India may fail to meet its installation targets on time. On the plus side, it presents an excellent opportunity to build domestic production capacity to fill supply chain gaps. Investigate potential revolutionary technologies that can assist India in meeting its solar targets at the same time.

Easy processing of Perovskite PV technology with better efficiency and wide availability of raw materials can be the solution. In this paper, we conducted a technoeconomic analysis to assess the commercial viability of perovskite solar cel (PSC) production in India. PSC technology has shown a substantial improvement in power conversion efficiency (PCE) from 3.8% in 2009^[^ ^16^ ^]^ to 25.6% in 2021.^[^ ^6^ ^]^ PSC's advantages include manufacturing processes such as screen‐printing known for use in the manufacture of conventional silicon solar modules; cost‐effective components (materials); and projected smaller manufacturing investments. PSC is a cost‐effective alternative to conventional and commercially available PV technology.^[^ ^17^, ^18^, ^19^ ^]^

The PSC has a different architecture than the conventional silicon solar cell. The basic architecture of PSC has a front and back electrode with an electron transport layer (ETL), a hole transport layer (HTL), and a sandwiched Perovskite absorber, as shown in Figure S1 in the Supporting Information.^[^ ^15^, ^16^, ^17^ ^]^ PSC counter‐electrodes may be either metallic (such as Au, Ag, etc.) or inorganic (such as carbon). They may be categorized as PSC metal counter‐electrode based PSC (MPSC) and carbon counter‐electrode based (CPSC).^[^ ^20^, ^21^, ^22^ ^]^ Metallic electrodes are typically incorporated in combination with organic HTLs and evaporated thermally, while carbon electrode based are generally screen printed. Most of the most efficient solar cells recorded to date have metal counter electrodes, whereas the carbon electrode‐based perovskite solar cells lead to lower cost with higher stability.^[^ ^23^, ^24^, ^25^ ^]^ CPSCs can be further classified as high temperature and low temperature processed solar cells. Both these carbon based architectures exhibits higher stability over other architectures. High temperature CPSCs proved one year stability over accelerated lifetime testing (IEC 61 215:2016)^[^ ^26^ ^]^ which is used for Si PV technology while low temperature CPSCs proved 5 years stability after dark storage in ambient condition.^[^ ^27^ ^]^

The high‐power conversion performance values of PSC captured tremendous market interest. Researchers and businesses have been actively interested in scaling the PSC from the laboratory to the modules. A detailed cost analysis study for optimizing cost drivers in estimating PSM's manufacturing costs must be carried out before the technology commercialization from laboratory research.^[^ ^28^, ^29^ ^]^ Usually, a technoeconomic study is performed for cost estimation and the study of possible cost‐management aspects. Resolving cost and performance challenges allows us to make timely decisions on technology commercialization. The cost estimates set the basis for the production set‐up costs and the computed unit cost, the minimum sustainable price (MSP) per unit, the representative lowest cost to sustain production financially, and govern the cost‐performance optimization. Various cost estimation techniques are available, such as expert judgment, three‐point estimation, analogous estimation, parametric estimation, bottom‐up estimation. The bottom‐up calculation methodology calculates the costs in the lowest possible detail, then summarizes the total costs. The bottom‐up calculations are slower but more precise than any other estimation technique.^[^ ^30^, ^31^, ^32^ ^]^

The literature on a cost estimate of the Perovskite Solar Cell Technology is relatively low compared to 19 794 papers on Perovskite Solar Cell Research (source: Web of Science). Recently, few technoeconomic research studies on certain PSM architectures have been reported. Asif et al. reported a technoeconomic assessment of PSM in 2015, assuming a 10% efficient 25 cm^2^ module, and concluded that PSM could never replace Si‐based solar modules.^[^ ^33^ ^]^ Later, Cai et al.^[^ ^34^ ^]^ compared two PSM architectures‐ high‐temperature carbon‐based PSM [FTO/c‐TiO_2_/m‐TiO_2_/ZrO_2_/C with CH_3_NH_3_PbI_(3 −_ *_x_*_ )_(BF_4_)*_x_*] and high‐efficiency, high‐precision PSM [ITO/PEDOT:PSS/perovskite/PCBM/Ca/Al]‐ and concluded that the cost of the module was $ 0.25–0.215 W^−1^ with module efficiency of 12–19%. The estimated costs indicated a lower manufacturing cost for CPSM relative to MPSM. Song et al. documented a manufacturing cost of $ 31.7 m^−2^ ($ 0.41 W^−1^) for p‐i‐n structured PSM (ITO/NiO/MAPbI_3_/ZnO/Al) using screen printing and Al sputtering at a 200 MW capacity manufacturing plant.^[^ ^28^ ^]^ The main assumptions were 16% PCE with 30 years lifetime. Chang et al. analyzed three architectures of glass‐based PSMs—FTO/c‐TiO_2_/perovskite/P3HT/Au; FTO/c‐TiO_2_/Perovskite/Poly(3‐hexylthiophene)(P3HT)/Ag; and FTO/c‐TiO_2_/Perovskite/P3HT/Silver—the modules estimated at $ 175, 102, and 90 m^−2^, the uncertainty analysis ranged manufacturing cost from $ 87–140 m^−2^ for a plant located in China.^[^ ^35^ ^]^ The study concludes that the LCOE of 9 ¢ kWh^−1^ can be achieved with 18% PCE and 20 years’ lifetime. In another publication, Chang et al. published a cost analysis for PSM employing roll‐to‐roll (R2R) manufacturing processes on ITO substrate with an estimated cost ranging from $ 37 to 74 m^−2^ at 10% PCE.^[^ ^36^ ^]^ Chang et al. used the bottom‐up technique in a reported analysis; the authors performed PSM cost analysis fabricated using a screen‐printed process along with evaporated Ag or Al rear electrode on flexible ITO substrates. Costing for five manufacturing flow combinations was analyzed alongside uncertainty analysis. Other assumptions include plant location as China, 15% PCE, 15‐year lifespan, and forecast $ 37 m^−2^ with key technology growth. Song et al. studied three architectures of PSM‐ single‐junction, two‐terminal, and four‐terminal all‐perovskite tandem—and estimated the MSP of $ 0.32–0.37 per W for PSM manufactured in the US.^[^ ^34^ ^]^ Li et al. reported four solar module configurations—Si PERC module, planar PSM, silicon/perovskite tandem module, and perovskite/perovskite tandem module with PCE of 21%, 19%, 25%, 22%. Estimated production costs were US$ 89.59, 32.69, 121.18, and 45.23 per m^2^ with estimated LCOE (¢ kWh^−1^) 5.5, 4.34, 5.22, and 4.22.^[^ ^37^ ^]^ In a recent paper, Ian et al. conducted a technoeconomic analysis assessing the impacts of economies of scale for PSM production of a single junction PSM built using R2R manufacturing and a perovskite‐silicon tandem solar cell. Flexible PSMs were estimated to cost $ 3.3–0.53 W^−1^ in 0.3 – 1000 MW per year production capacities.

Perovskite solar cell research is a dynamic field. Researchers are developing various materials, processes, and architectures that can influence the efficiency, manufacturing cost, and, in turn, PSM's viability.^[^ ^38^, ^39^ ^]^ The development of low temperature processed carbon perovskite solar cells is one such technology that drives CPSM costs. Most of the cost estimation literature is focused on MPSM because of their reported higher efficiencies compared to CPSM. However, they pose processing constraints, instability issues, and expensive materials compared to CPSM.^[^ ^40^ ^]^ The cost estimation is primarily influenced by the selected PSM architecture, chosen materials, planned manufacturing processes, plant capacity, assumed efficiency, and plant location.

This paper focuses on applying bottom‐up cost estimation techniques to CPSM manufacturing in India, an emerging photovoltaic manufacturing hub.^[^ ^11^ ^]^ Two typical CPSM architectures, high‐temperature processed CPSM (FTO/c‐TiO_2_/m‐TiO_2_/ZrO_2_/C) [41,42] and low‐temperature processed CPSM (FTO/c‐SnO_2_/Perovskite/NiO*_x_*/C),^[^ ^43^, ^44^, ^45^ ^]^ were thoroughly analyzed for cost estimates. The primary process in CPSM is assumed to be screen printing. The analyzed 100 MW capacity manufacturing plant is presumed to be located in Himachal Pradesh, India. Considering 12.77%^[^ ^46^ ^]^ and 13.57%^[^ ^47^ ^]^ efficient modules (75% of reported maximum laboratory efficiencies), the manufacturing cost estimate, the minimum sustainable price estimate, was carried out. Sensitivity analysis was conducted around material costs and other operating parameters. The LCOE is estimated based on a 10‐year and 25‐year module lifetime. According to our knowledge, this is the first study to evaluate manufacturing cost of low temperature processed carbon‐based PSM, evaluating the feasibility of perovskite solar cell manufacturing in emerging markets, including initial investment, calculation of the minimum subsistence price of generated modules, and levelized energy cost produced in India using these modules.

## 2 Methodology

**Figure 1:**
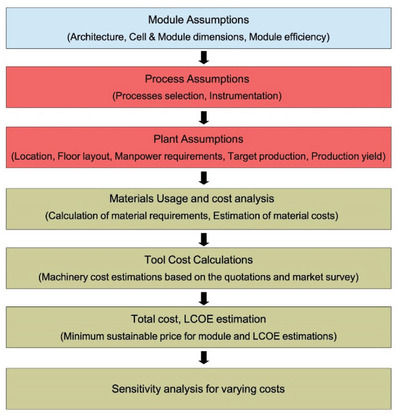
Flow of technoeconomic analysis calculation for CPSC.

Costing analysis of both the modules was performed for two carbon‐based perovskite modules, i.e., high temperature (HT) and low temperature (LT); using bottom up technique. For this purpose, it was assumed that manufacturing plant is located in Himachal Pradesh Baddi and will start to operate from 2022. A plan of 100 MW yearly production with corresponding cost analysis is projected and bottom up technique was utilized for detailed calculation. Bottom‐up is the comprehensive cost calculation technique that helps in precise cost calculation. Various studies suggest that Bottom‐up is a suitable technique for better cost estimation of an emerging PV technology.^[^ ^29^, ^32^, ^35^, ^42^, ^48^ ^]^ Costing analysis was conducted by dividing the entire CPSC module production into various categories from module assumption to material use, tool costing, plant location, as shown in **Figure** **1**.

The hypothesis and all the assumptions began with the module size, followed by processes leading to plant‐related assumptions that led to a cost analysis of the material and manufacturing costs. The minimum sustainable price (MSP) is calculated by summing up both direct and indirect costs, including material costs, utility costs, labor costs, operating costs, depreciation costs, borrowing interests.

### 2.1 Assumptions

#### 2.1.1 Module Assumptions

**Figure 2:**
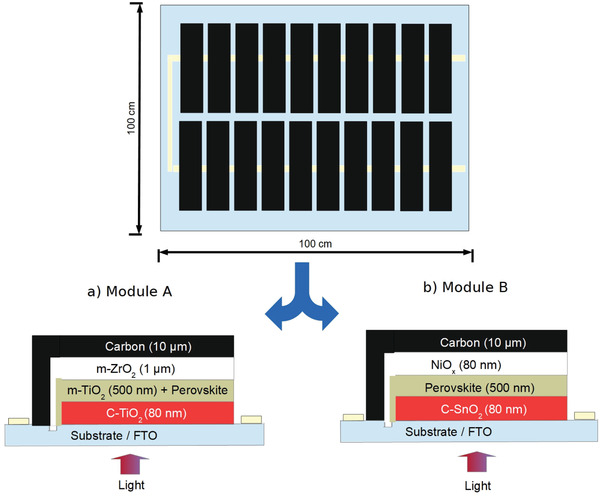
Carbon‐based Perovskite module (CPSM) architectures a) Module A: conventional high temperature processed CPSM b) Module B: Low temperature processed CPSM.

The costing study was performed for two different geometries, i.e., processed high temperature and processed low‐temperature modules (as shown in **Figure** **2**) based on rigid glass‐based geometry with “n‐i‐p” configuration. These geometries and materials are preferred because they include low‐cost materials and are reported as highly efficient with better stability on a laboratory scale.

Glass‐glass module configuration is presumed to retain power conversion efficiency (PCE) of 75% of its highest lab recorded cell efficiency. The efficiency drop from lab scale to the manufacturing scale is also observed in other thin‐film solar cell technologies such as CIGS. Each PSM is designed to be 1 square meter with 20 monolithically integrated cells. This concept of monolithically integrated solar cells offers various advantages including all contacts on same substrate, faster processing with reduced post solar cells production processes including soldering contacts.^[^ ^49^ ^]^ A detailed pictorial description of module design, corresponding architectures is shown in Figure 2. The particulars about these modules such as module design, performance, and peak capacity etc., are described in **Table** **1**. It is observed that high *T* module (A) and low *T* module (B) have different material requirements with huge difference in temperature requirement of 500 and 150 °C. The highest reported solar cell efficiencies for solar cell architectures A and B are 17.02%^[^ ^46^ ^]^ and 18.1%^[^ ^47^ ^]^ respectively. Considering screen printing to be the common technique utilized in both architecture we performed the material selection accordingly. As scaling up of solar cells to modules results in various losses, thus we considered the module efficiency to be 75% of the cell efficiency. The assumption of 75% is consistent with the reported drop in efficiency from lab scale devices to large area devices by Rong et al.^[^ ^50^ ^]^ In reality the drop may vary based on many factors including series resistance, recombination effects due to interfaces, film morphology etc.

**Table 1:** Parameters of the designed module

	Module A	Module B
Module architecture	FTO/c‐TiO_2_/m‐TiO_2_/ZrO_2_/Carbon (perovskite infiltrated)	FTO/c‐SnO_2_/Perovskite /NiO*_x_*/Carbon
Maximum processing temperatures	500 °C	150 °C
Reported lab‐scale cell efficiency	17.02%^[^ ^46^ ^]^	18.1%^[^ ^47^ ^]^
Module efficiency (75% of cell efficiency)	12.77%	13.57%
Module size	1 m^2^	1 m^2^
Distance between cells	0.2 cm	0.2 cm
Margin areas at left and right sides of the module	0.9 cm	0.9 cm
Margin areas at up and downsides of module	0.5 cm	0.5 cm
The gap between two parallel (10 cells) row	0.5 cm	0.5 cm
Size of rectangular cells	48.85 cm X 9.74 cm	48.85 cm X 9.74 cm
Number of cells on one piece of module	20	20
The power output of one piece of module	127.7 W	135.7 W

Beside these other parameters such as distance between cells, no. of cells etc. for the module fabrication were considered the same for both modules. As architecture selection, fabrication procedure directly affects the throughput of manufacturing plant as reported by Cai et al..^[^ ^33^ ^]^ Following this to obtain the yearly target of 100 MW production from the manufacturing plant, power of module plays a major role. The relation between module efficiency and power was utilized for this purpose.^[^ ^33^ ^]^ A direct relation between module efficiency and corresponding power generation was observed for both modules. Higher efficiency module B exhibits more power generation over module A. Thus, leading to requirement of lesser modules B production than module A. The PSMs are further processed and sealed using the essential balance of materials (BOM) as used in silicon solar module production. BOM components include glass plates, lamination film, edge sealants, bus bars, wiring, and junction box. The detailed material bill is designed, including all the essential components, including the junction box, wiring, bus bars, sealants, back sealing substrate, etc.

#### 2.1.2 Process Assumptions

**Figure 3:**
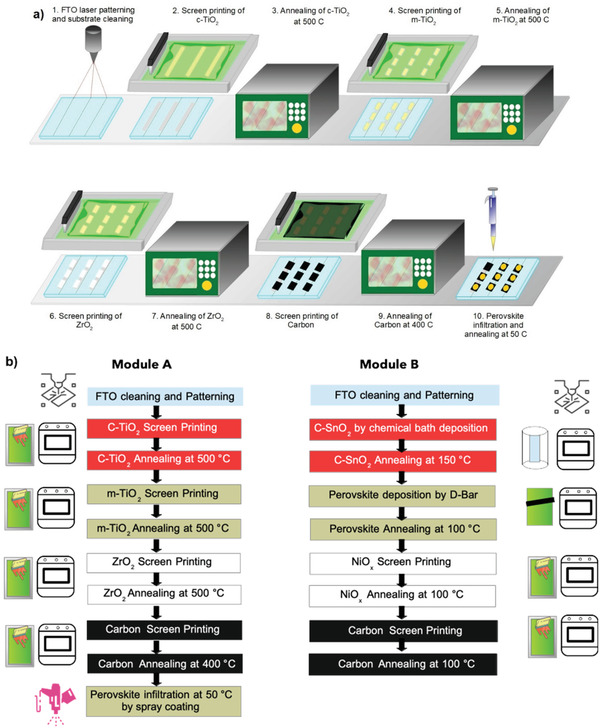
a) Schematic of Module A production layout b) Fabrication process flow chart of module B.

A step‐by‐step batch process layout for production of mesoporous high temperature module A is designed as shown in **Figure** **3**a followed by detailed description of fabrication of both modules in Figure 3b. From this it was observed that screen printing is the most widely used technique for production of both modules with variation in perovskite deposition processes. Advantages such as easily scalability with high throughput in ambient processing conditions results in lesser requirement of skilled labors and thus solves the costing issues in module production. Utilization of highly reproducible and cost‐effective screen‐printing technique points towards the easy installation of such manufacturing setup worldwide with lower investment.

Module A comprises compact TiO_2_, meso‐TiO_2,_ and ZrO_2_ layers screen‐printed and processed at high temperature (≈500 °C) followed by screen‐printed carbon electrode, then annealed at 400 °C. Various reports corresponding to perovskite infiltration using drop‐casting, robotic dispenser, inkjet infiltration, and slot die coating are reported. These processes are briefly described in detail by Meroni et al., Hashmi et al., and Xu et al..^[^ ^34^, ^51^, ^52^ ^]^ Among them, drop‐casting in combination with nozzle spray is selected for infiltration of perovskite with composition (5‐AVA)*_x_* (MA)_1‐_ *_x_* PbI_3_ followed by annealing of infiltrated devices at 50 °C.

In contrast, the first layer of SnO_2_ in low temperature processed module B is deposited by chemical bath deposition technique followed by annealing at 150 °C. D‐bar coating was then used to deposit the perovskite layer, followed by NiO*_x_* deposition (HTL) and low *T* carbon electrode by a screen‐printing technique. Koet al., Jeong et al., Song et al., Ye et al. and Kajal et al. reported the low temperature fabrication processes of different layers.^[^ ^28^, ^53^, ^54^, ^55^, ^56^ ^]^ The type of module selection changes the machinery, investment with production time and production layout. Manufactured modules are then designed to encapsulate using a glass‐glass arrangement followed by a junction box installation. Module testing is the final step before storage/dispatch.

#### 2.1.3 Manufacturing Plant Assumptions

A 100 MW manufacturing plant is anticipated to be located in Himachal Pradesh, India. Two parallel production lines were assumed for module A, with a 50 MW per year production capacity for each line. Manufacturing plant assumptions include plant location, electricity, construction costs, service electricity, floor space ratio, facility depreciation, plant hours, and skilled labor availability. A 3.0‐acre manufacturing plant with a building area of 23 sq. meters MW^−1^ and a lifetime of 10 years is scheduled to start operations from 2022 with a construction duration of 12 months. The manufacturing plant financing is planned with debt and equity in the proportion of 70:30. The interest rates for debt and cost of capital for equity are accounted to be 9% and 13%, respectively. The total weighted average cost of capital cost (WACC) is assumed to be 10.2% with an NPV rate of 10% with land, labor, and utility prices by location. A detailed description about assumed parameters of the manufacturing plant is given in **Table** **2**.

**Table 2:** Manufacturing plant assumptions

Parameter	Assumption	Parameter	Assumption
Factory location	India (Himachal Pradesh)	Cost of the building (million US$)	0.85
Factory start date	2022	Cost of P&M (million US$)	7.51
Depreciation time‐ equipment and facilities	10 years	No. of employees	150 (A) and 100 (B)
Depreciation time‐ building	20 years	Cost of labor (million US$ year^−1^)	85.23 with inflation of 5%
Currency for calculation	US$	Dominant manufacturing technique	Screen Printing
Electricity cost (US$ kWh^−1^)	0.07 with energy inflation 3%	Material utilization	80%
Yearly objective	100 MW per year	Mature or immature technique	Mature technique so highly skilled workers are not required
Land requirement	3.0 acre	Plant operation time (h day^−1^)	16 h day^−1^
Building area	23 sq meters MW^−1^	SG&A, R&D, and overhead cost	10.23 US$ ( MW^−1^)
Cost of land (million US$)	0.2	Discount rate (WACC)	10.2%
Depreciation type and rate	5.83% for book value SLM; 5% for building; 12% Plant and machinery; 5% for other assets	Throughput (Modules per day)	Module A: 3262 Module B: 3070

### 2.2 Cost Analysis

Costs are split into capital and operating costs. Capital costs involved in CPSM production include land costs, construction costs, plant and machinery (P&M) costs. Tables ST1 and ST2 in the Supporting Information display descriptions of the machines needed, their cost and floor requirements, their power requirements for modules A and B. Plant and Machinery costs and building costs are specified in Table 2. Capital costs are distributed as interest and depreciation of fixed costs. The manufacturing plant was assumed to depreciate linearly in 10 years. In this article, for ease of analysis, we assumed that raw material costs were stable. Five percent and three percent inflation are assumed for labor and energy, respectively. Consequently, the cost of producing modules can be measured by adding up all the costs as indicated in Equation (1)

(1) $$MC\;\;=\;\;\underset{i}{\sum }\;\;\left(M_{i}+U_{i}+L_{i}+E_{i}+D_{i}\right)$$

where *M_i_* is material cost; *U_i_* is utility cost (including electricity and water); *L_i_* is labor cost; *E_i_* and *D_i_* is equipment maintenance cost and depreciation cost including building, equipment respectively in the i^th^ step of the process.

Labor costs are determined by the location of the manufacturing plant and the operating time of the plant. Still, the cost of materials and utilities is directly influenced by the module architecture and production processes. Below, a brief description of all applicable costing criteria is discussed.

#### 2.2.1 Material Cost (*M_i_*)

Two factors, namely the amount of material used and the unit material's cost, determine the perovskite module's material cost.^[^ ^29^ ^]^ The material requirement is calculated by using screen printing of pastes for a smaller area. The material's weight is measured for a smaller area (cell area) and then multiplied by the required module area to calculate for the module. However, the D bar coated perovskite layer material usage is calculated using the density (ρ) and thickness (*t*). It is assumed that the material waste is 10%. A detailed calculation of material requirement for module production with 10% material wastage is shown in Table ST3 and ST4 in the Supporting Information for modules A and B, respectively. Total material cost (M) is typically derived by multiplying the quantity of material used (MU) with its unit cost. The unit cost of different materials was collected through various databases, including published literature, quotations from various manufacturers, and e‐commerce websites.

#### 2.2.2 Utility Cost (*U_i_*)

Utilities primarily cover the cost of electricity for the building of such a plant. The energy usage is calculated by summing up each piece of equipment's power consumption from the power rating and utilization factor. It is estimated that the total energy consumption is ≈7659.87–12 938.31 kWh per MW of production. This model assumes an electricity cost of US$ 0.073 kWh^−1^ with annual inflation of 3%.

#### 2.2.3 Manpower (Labor) Costs (*L_i_*)

Considering literature reported cell architectures, the modules are believed to be primarily created by screen printing. It then contributes to the manufacturing plant needing non‐skilled laborers. A total of 150 and 100 employees were assumed at each printing site of module A and B respectively, for the semi‐automatic plant with a minimum of 1 labor. Labor costs are projected w.r.t to plant in Himachal Pradesh, India, with an inflation rate of 5%. The information relevant to all these parameters is provided in Table ST3 (Supporting Information).

#### 2.2.4 Equipment Maintenance Costs (*E_i_*)

Real maintenance costs to the company include lost sales due to repairs, production costs incurred during maintenance. As an industry‐standard, 1% of the total capital cost is defined as the plant's maintenance cost per year. This article assumed maintenance costs equivalent to industry standard (1%).

#### 2.2.5 Depreciation Costs (*D_i_*)

Depreciated costs are the value of a fixed asset, minus all cumulative depreciation. An industry‐standard assumes a 5% depreciation rate for buildings, 12% for plants and equipment, and 5% for other assets.

## 3 Results

### 3.1 Manufacturing Cost

**Figure 4:**
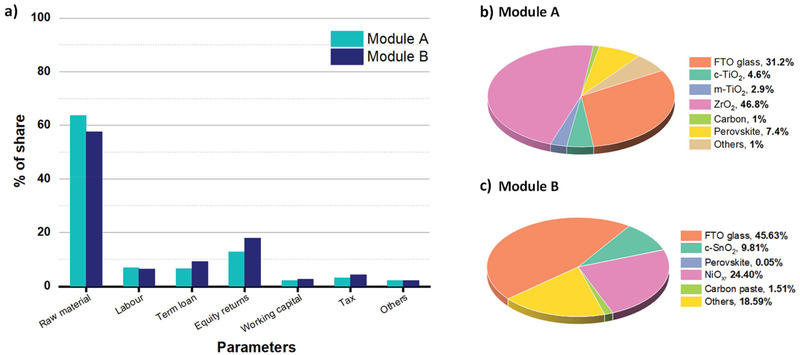
a) Different parameters and their share analysis for both modules, b) material share for module A, and c) material share for module B.

Manufacturing costs of solar modules (US$ W^−1^) were calculated by adding materials, manufacturing, and other critical parameters cost in Equation (1). **Figure** **4**a–c shows the components individual costs of both modules shown in Figure 2. The average production cost for Modules A and Module B are US$ 0.21 per W and US$ 0.15 per W, respectively.

Material costs account for a large share of the total cost, contributing 63.7% and 57.55% of total cost in Module A and B. ZrO_2_, a scaffold layer in Module A, contributes the most considerable material cost difference in Module A and Module B. The high cost of ZrO_2_ contributed 46.8% of the material cost for Module A and is considered one of the susceptible factors towards variation in the module cost. Other material parameters of Module A, i.e., FTO, compact TiO_2_, meso TiO_2_, carbon, and perovskite share 31.2%, 4.6%, 2.9%, 1%, 7.4% with 1% share of others (including solvents, cleaning materials, BOM, etc.). Similarly, low‐temperature module B with 57.55% of material share composed of FTO, ETL, Perovskite, HTL, Carbon, and others as 45.63%, 9.81%, 0.05%, 24.4%, 1.51%, and 18.59% respectively, as shown in Figure 4a–c.

Followed by material costs; capital cost also plays a significant role in deciding production cost. For modules A and B, the aggregate of equity returns, term loans, and working capital contributes 22.08% and 29.68%. With high inflation, India's cost of capital exceeds many other countries. The capital cost can vary depending on location; lower costs of capital would further reduce production costs. However, that will also influence the labor costs. The sales‐related costs and total costs of research and development have a minimal contribution in the range of 1.18% and 1.87% for Module A and B.

Depending on lower land costs and easy labor access, the evaluated PSM's production costs in this study are lower than previously recorded module costs. This is confirmed by comparison with various reports, including Cai et al. predicted the high *T* carbon module (module A) cost US$ 0.25–0.28 per W; and the Au module cost around US$ 0.21 – 0.26 per W ^[^ ^43^ ^]^ and MSP of US$ 0.41 W^−1^ by Song et al.^[^ ^22^ ^]^ for screen printed conventional perovskite solar cells; with manufacturing units located in China and USA respectively.

With a minor variance in processing facilities and temperature specifications, productivity level varies slightly. Given their higher processing temperature requirements, around 3262 Module A's can be produced, and at the same time, approximately 3070 module B's can be built.

#### 3.1.1 Net Present Value

A discounted cash flow or Net Present Value (NPV) based accounting model has been used to calculate the module manufacturing costs. NPV model uses the following Equation (2) as

(2) $$\begin{array}{c} \text{NPV}\;\;=\;\;\underset{t}{\sum }\frac{R_{t}}{{\left(1+i\right)}^{t}}\;\;-\;\;\frac{C_{t}}{{\left(1+i\right)}^{t}}-I_{o} \end{array}$$

Where *R_t_* = flow of expected revenues for the time period *t*, *C_t_* = flow of costs incurred during the time period *t*, *i* is discounting rate, and *I*_0_ is an initial level of investment (fixed capital cost). This method has proposed the linear depreciation cost to distribute cost uniformly, comprising an income tax rate of 25% with a minimum alternate tax rate of 21.34%. The outcome of the models is discussed in subsequent sections.

#### 3.1.2 Minimum Sustainable Price (MSP)

MSP is defined as the minimum sustainable price at which the Internal Rate of Return (IRR) of a PV manufacturing plant is equal to the weighted average cost of capital (WACC). It depends on the PCE of the PV module and is written as Equation (3)

(3) $$\begin{array}{c} \text{MSP}\;\;=\;\;\left(\text{MC}+\text{OH}+\text{WACC}\right)/\left(\eta *Po\right) \end{array}$$

Hence, the MSP of a solar module comprises manufacturing cost, overhead cost (sales, general and administrative cost), and the weighted average cost of capital. MSP analysis was performed with a WACC of 10.2% at an NPV rate of 10.0%. The manufacturing plant had a 10 years lifetime with 1% OH expenses, 0.4% insurance cost, and 1% S and G, R and D costs was assumed with a total capital utilization of 70%. A detailed MSP analysis for cost variation w.r.t. PCE was performed for the financial parameters shown in **Table** **3**.

**Table 3:** MSP assumptions

Assumptions	A	B
Plant capacity (MW)	100	100
Total land area (acre)	3	3
Equipment cost (US$ million)	7.51	7.51
Human resources	150	100
Debt: Equity	70:30	70:30
Life of the plant (years)	10	10
Construction period (months)	12	12

**Figure 5:**
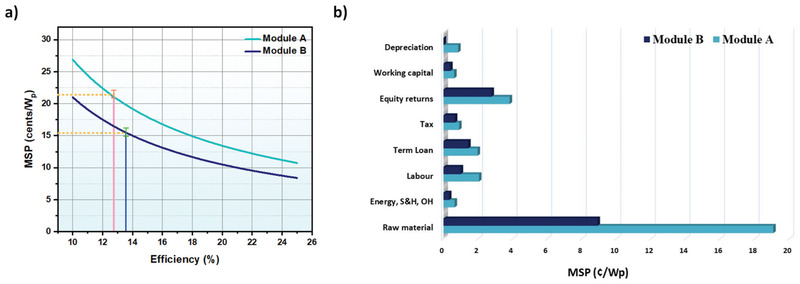
a) MSP calculation for module A and B with variation in module efficiency. b) Comparative analysis between different MSP components.

Considering the plant's target of manufacturing 100 MW in 12 months, a 3‐acre land area with two parallel operating lines is presumed. This objective was solved by integrating machines with at least one operator at each location, and official workers, i.e., 150 and 100 human resources, are assumed to manufacture of module A and B respectively. As for a manufacturing plant's continuous operation, solar modules’ selling price should meet the MSP value. In this study, we assumed the solar PV manufacturer in India and achieved an MSP of US$ W^−1^ of 0.21 and 0.15 for modules A and B, respectively. The difference in MSP as a function of module efficiency (i.e., PCE of 12.77% and 13.57% for module A and B) is shown in **Figure** **5**.

Figure 5a) indicates the MSP variation corresponding to the module's efficiency with an increment of 0.5% in PCE. An exponential reduction in MSP is observed for the increment of PCE from 10% to 20%. A PCE of 10% modules A and B exhibits an MSP (cents per W_p_) of 26.86 and 20.99, respectively, suggesting a huge gap in the MSP for both modules. A huge reduction in the MSP value was observed with an increment in PCE to 25%. An MSP (c W^−1^) of 10.74 and 8.39 is observed for modules A and B, respectively.

Other factors contributing to both modules’ MSP variation are shown in Figure 5b along with manufacturing technique and material variation. The main parameters are thus classified as raw materials (R), energy S and G OH (ESGO), labor (L), term loan (TL), tax (T), equity returns (E), working capital (W), and depreciation (D). These parameters are calculated for the assumed PCE of 12.77% and 13.57% for modules A and B. Module A with an MSP of 21.07 c W^−1^ at a PCE of 12.77% consists of 13.84 R, 0.37 ESGO, 1.49 L, 1.43 TL, 0.66 T, 2.78 E, 0.45 W, and 0.07 D. While Module B exhibits an MSP of 15.48 c W^−1^ at a PCE of 13.57% with ≈8.91 R, 0.32 ESGO, 0.99 L, 1.43 TL, 0.66 T, 2.77 E, 0.39 W, and 0.01 D. This indicates that Module A has more MSP due to more R, ESGO, W and D over module B.

#### 3.1.3 Sensitivity Analysis

**Figure 6:**
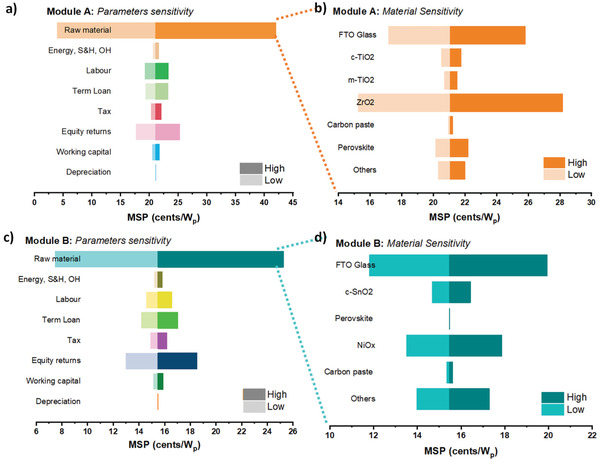
Sensitivity analysis for 10% deviation in MSP for a,c) module parameters cost and b,d) material cost.

As MSP plays a significant role in deciding a module's actual cost. Considering variations between assumed and actual value, their modeling assumptions would directly impact MSP. Therefore, sensitivity analysis for both modules is performed. A sensitivity analysis explains how various values of an independent variable impact the outcome under certain conditions. The study examines the contribution of multiple sources of uncertainty to a mathematical model's overall vulnerability. Perovskite solar modules’ most essential variables are performance and material costs. Conversion efficiency and solar cell costs have an inverse relationship. This study studied two distinct architecture styles. Modules A and B were thought to have an average efficiency of 12.77% and 13.57%, respectively, 75% of the cell‐level efficiency stated in the literature. A sensitivity analysis was performed to assess the minimum sustainable price's impact with 10% price deviation of each variable, as shown in **Figure** **6**. As the material is the most contributing factor in the cost of both modules. Hence sensitivity analysis is performed based on each parameter variation a, c) and material in Figure 6b,d. 10% deviation in both increasing and decreasing cost is analyzed, and for calculating its impact on MSP, where MSP is considered as the middle term.

From Figure 6a,c, it's been found that all raw material is the most sensitive component in MSP for both modules. Other than materials, Module A MSP, shows sensitivity characteristics to E and L in Figure 6a. Module B cost shows higher sensitivity characteristics to R, L, TL, T, E, W; indicating modules B being the low temperature and low cost but are very sensitive to market fluctuations.

As raw material cost is the most sensitive parameter in the module cost. Thus, a sensitivity analysis is performed on the basis of materials utilized. This material‐based sensitivity analysis would help in depicting the component playing crucial role in varying the cost of modules. This can also provide the target to research community to search for alternating cost‐effective solutions to reduce the cost without compromising efficiency.

From the material sensitivity of Module A in Figure 6c, it is observed that ZrO_2_ is the most contributing factor towards the main cost for module A. 10% deviation in cost of ZrO_2_ paste will affect the MSP to a more considerable extent. Similarly, in module B, material sensitivity analysis in Figure 6d FTO, ETL, HTL, and other components are the most sensitive components; cost variation of these materials will affect the main module cost to a more considerable extent. Also, sensitive components in module B material cost more than module A.

But the obtained MSP of the carbon module is lower than conventional metal electrode‐based architectures. Lower MSP for carbon‐based modules is due to simple instrumentation, non‐sophisticated equipment, low‐cost material involving unskilled labor. Lower MSP of carbon‐based perovskite solar cells is linked to lower sales price regardless of the marketplace, process advancement, or competitiveness with simplistic production sequence.

#### 3.1.4 Levelized Cost of Energy (LCOE)

LCOE is defined as minimal electricity production costs and is calculated for assessing different sources of power generation. The electricity sector commonly uses the term to analyze the solar electricity cost. LCOE provides information about the economic competitiveness of different electricity generation technologies.^[^ ^30^, ^31^, ^32^, ^33^ ^]^ It can also be defined as the ratio of the total lifecycle cost of the PV system to the total energy generated during the lifetime of the PV system and is calculated using Equation (4) as

(4) $$\begin{array}{c} \text{LCOE}\;=\frac{\text{Sum}\;\text{of}\;\mathrm{costs}\;\text{over}\;\text{lifetime}\;\text{Sum}\;\text{of}\;\text{electricity}}{\text{energy}\;\text{produced}\;\text{over}\;\text{lifetime}}\;=\frac{\sum_{t=0}^{n}\frac{C_{t}}{{\left(1+r\right)}^{t}}}{\sum_{t=0}^{n}\frac{E_{t}}{{\left(1+r\right)}^{t}}}\; \end{array}$$

where C_t_ is the total cost in the t^th^ year, and *E*_t_ are energy production (kWh) in the t^th^ year; r is a discount rate, and n is the lifetime of the system.

**Figure 7:**
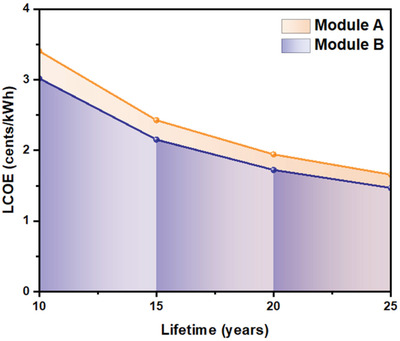
LCOE of carbon‐based perovskite modules A and B with average PCE of 80% with a lifetime of 10, 15, 20, and 25 years.

This section estimated LCOE for two widely reported carbon‐based perovskite solar cell technologies with an assumption of the module's lifespan of 10 years. The average electricity generation of the Perovskite module‐based power generation plant is estimated to be 100 MW with annual system costs as shown in Equation (5)

(5) $$\begin{array}{c} Ct\;\;=\;\;I_{t}+O_{t}+F_{t} \end{array}$$

where I_t_ is the initial installation cost, and it consists of the initial cost of PV modules, wiring cost, the balance of system cost, land fees, taxes, and overhead cost, which is considered for the initial year. O_t_ is the system operating cost, and *F*_t_ is financing cost of year “*t*” including tax, insurance, incentives, etc. The LCOE for both Carbon‐based perovskite modules were calculated using MSP values as shown in **Figure** **7**. **Tables** **4** and **5** provide input values for LCOE calculations. LCOE calculation consisted of module efficiency, lifetime and solar insolation on selected location. 12.77% and 13.57% is assumed to be PCE of module A and B respectively with 75% reduction from cell efficiency. Assumed modules were then subjected to analysis of lifetime at 10‐ and 25‐years gap by maintaining 80% PCE than initial efficiency. PV installation cost is then calculated from these assumptions.

**Table 4:** module parameters for LCOE calculation

Parameter	Module A	Module B
Initial efficiency	12.77%	13.57%
Lifetime	10, 25 years	10, 25 years
Annual average daily solar Insolation (kWh m^−2^)	4.5	4.5

**Table 5:** PV system installation cost inputs for benchmark LCOE calculation

Input parameter	Module A [$ W^−1^]	Module B [$ W^−1^]
Perovskite PV module	0.21	0.15
Balance of system (land, etc.)	0.28	0.28
Total installed cost	0.49	0.43

The high T and low *T* modules were assumed to be fabricated with an installation cost of US$ W^−1^ 0.50 and US$ W^−1^ 0.43. The PV plant is believed to be located in Himachal Pradesh, India, with an annual average irradiance of 4.5 sun‐hours per day per year. In addition to examining the effect of plant life on LCOE, cases were also investigated for 15, 20, and 25 years. The LCOE obtained includes installation, running, and maintenance costs given by Himachal Govt (REF). LCOE of module ranges from US$ 0.034to 0.016 kWh^−1^ for module A and US$ 0.030–0.015 kWh^−1^ for module B with frequency differences varying from 10% to 25%.

**Figure 8:**
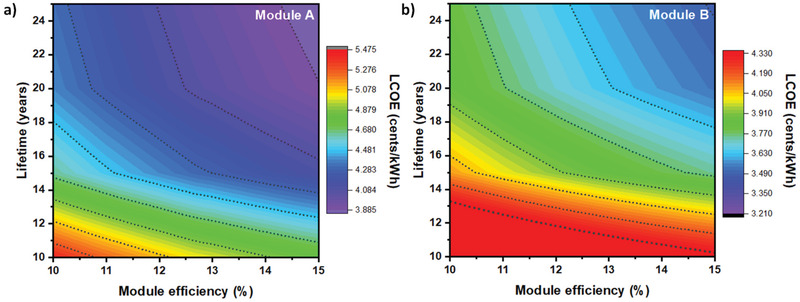
LCOE calculation for carbon modules using relation between efficiency and lifetime with a degradation rate of 1% per year for a) module A and b) module B.

As module efficiency, degradation rate and lifetime are very important components for predicting the LCOE of modules. Thus, using on module efficiency variation (10–15%) and lifetime from 10 to 25 years with the assumed degradation rate of 1% per year the LCOE analysis is calculated for both modules as shown in **Figure** **8**a,b. These shows that high PCE modules with larger lifetime leads to lower cost. As module A exhibit higher MSP than module B leading to higher LCOE of module A over B indicated by dotted lines.

## 4 Conclusions

There is a lot of talk about the economic viability of perovskite solar cells. India's supply chain disruption in the solar PV industry has opened up new generation solar cell technology opportunities. The minimum sustainable price for a carbon‐based perovskite module manufactured in India would be 21.06 and 15.48 ¢ W^−1^ for different module architectures. Hence, the material costs majorly drive the cost; variation in material cost will affect the module cost in the future. Sensitivity analysis for material cost and other parameters cost is calculated with a 10% standard deviation from MSP. Module B was observed to be highly sensitive to most of the parameters than module A. LCOE calculation for both modules was performed corresponding to the lifespan of 10, 15, 20, and 25 years maintaining 80% of PCE. LCOE varies from US$ 0.034 to 0.016 kWh^−1^ for module A and US$ 0.030–0.014 kWh^−1^ for module B, with lifetime variation from 10 to 25 years while maintaining 80% of initial PCE at corresponding year.

This study will help policymakers, investors, and other stakeholders make informed decisions about implementing this technology. The analysis has been done on a smaller production capacity where operational stability of low *T* CPSCs is assumed to be equivalent to high *T* CPSCs with constant degradation rate of 1% over time. Both the modules were fabricated using screen printing technology which results in lower geometric fill factor (g‐FF). As efficiency and GFF are basic components during commercialization of any PV technology.^[^ ^57^, ^58^ ^]^ Thus GFF can be further enhanced by coating whole substrate uniformly with a material layer and then usage of laser technique to separate those regions. Along with this monolithic fabrication of solar cells is assumed, which leads to faster module processing. However there are certain challenges associated with size and number of monolithic arrangements such as poor performance due to introduction of additional parallel resistance to the cell which leads to shift in Voc of the module and thus performance. Also during hot spot condition its difficult to neglect the effect of shunted cells from module.^[^ ^49^ ^]^ Thus considering the advantages and flaws associated with monolithic arrangement, the size and no. of cells should be carefully chosen. These, factors and economies of scale will help to bring the costs down. The technoeconomic framework helps researchers assess the feasibility of similar emerging technologies or this technology with the updated process, efficiency, stability and raw material prices in the future.

## Conflict of Interest

The authors declare no conflict of interest.

## Supporting information

Supporting Information

## Data Availability

Research data are not shared.
