# Supplementary material for: Costing Analysis of Scalable Carbon‐Based Perovskite Modules Using Bottom Up Technique
Source: Glob Chall. 2021 Oct 31;6(2):2100070. doi: 10.1002/gch2.202100070 (PMC8812919; doi:10.1002/gch2.202100070)
Supplement: Supplementary file 1 — Supporting Information [file GCH2-6-2100070-s001.pdf]

Global Challenges

Supporting Information

for *Global Challenges,* DOI: 10.1002/gch2.202100070

Costing Analysis of Scalable Carbon-Based Perovskite Modules Using Bottom Up Technique

*Priyanka Kajal, Bhupesh Verma, Satya Gangadhara Rao Vadaga, and Satvasheel Powar**

Supplementary Information

Costing analysis of scalable carbon-based perovskite modules using bottom up technique

Priyanka Kajal[a], Bhupesh Verma[b], V. S. Gangadhara Rao[c], Satvasheel Powar[a,d,*]

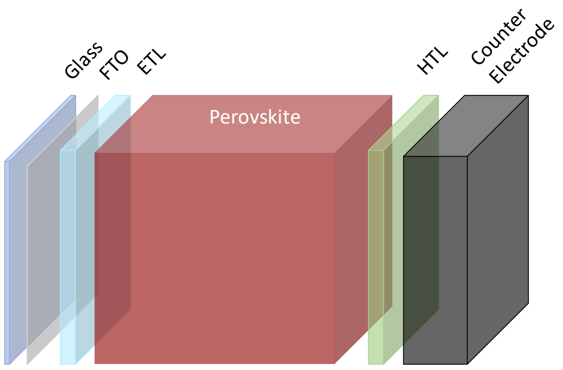

Fig S1: General architecture of carbon based perovskite solar cells

Table ST1 Machinery required for manufacturing plant of module A

S.No.	Machine	footprint (m*m)	Cost (USD)	Power (kW)	operating time (min/module)	No of machinery required	Source
1	Laser scriber	15	59206.17	2	0.25	1	India Mart (https://www.indiamart.com/)
2	screen printer	15	7776.68	10	0.17	4	Quote by SK Ensure machinery
3	Furnace	15	9922.82	30	1	4	India Mart (https://www.indiamart.com/)
4	Ultrasonic bath	6	99968.25	28	1	2	India Mart (https://www.indiamart.com/)
5	solar laminator	15	39645.20	35	1	1	India Mart (https://www.indiamart.com/)
6	Soldering system	12.5	128271.09	10	0.3	1	India Mart (https://www.indiamart.com/)
7	solar testing machine	2.5	12333.76	0.5	0.2	1	India Mart (https://www.indiamart.com/)

Table ST2 Machinery required for manufacturing plant of module B

S.No.	Machine	footprint (m*m)	Cost (USD)	Power (kW)	operating time (min/module)	No of machinery required	Source
1	Laser scriber	15	59206.17	2	0.25	2	India Mart (https://www.indiamart.com/)
2	Ultrasonic bath	15	99968.25	28	1	1	India Mart (https://www.indiamart.com/)
3	screen printer	15	7776.68	10	0.17	1	Quote by SK Ensure machinery
4	D coater	6	2885.41	10	0.25	2	India Mart (https://www.indiamart.com/)
5	solar testing machine	15	12333.76	0.5	0.2	1	India Mart (https://www.indiamart.com/)
6	solar laminator	12.5	39645.20	35	0.5	1	India Mart (https://www.indiamart.com/)
7	Furnace	2.5	9922.82	30	60	1	India Mart (https://www.indiamart.com/)
8	Soldering system	12.5	128271.09	10	0.3	1	India Mart (https://www.indiamart.com/)

Table ST3: Material requirement and corresponding cost for module A

Component	Parts	Units	usage+ 10 wt % wastage	Material Cost (USD/module)	Source
FTO Glass	conductivity > 7 per square	m^2	1	55.13	India Mart (https://www.indiamart.com/)
FTO cleaning	liquid soap	ml	11	0.15	Quote by Jaiswal chemicals
	DI water	ml	11	0.15	Quote by Jaiswal chemicals
	Ethanol	ml	11	0.38	Quote by Jaiswal chemicals
c-TiO_2 (screen printing)	Blocking layer TiO_2	g	7.7	8.07	Great cell solar
m-TiO_2 (screen printing)	18 NRT TiO_2 ink	g	1.79	5.07	Great cell solar
ZrO_2 (screen printing)	ZrO_2 paste	g	5.43	82.62	Solaronix
Carbon paste (screen printing)	ink	g	14.38	1.82	India Mart (https://www.indiamart.com/)
Perovskite (drop casting)	MAI	g	11.53	7.89	Quote by Jaiswal chemicals
	PbI_2	g	33.28	1.97	Quote by Jaiswal chemicals
	DMF	g	60.5	0.38	Quote by Jaiswal chemicals
	AVAI	g	0.67	2.86	Quote by Jaiswal chemicals
Solder wire	Sn	g	1.07	0.01	[1]
Edge seal	Al	g	1555	3.84	[1]
Lamination	EVA	g	138	1.51	[1]
Edge sealant	butyl rubber	g	10.5	0.04	[1]
Back glass	2mm glass	m^2	1	2.96	[1]

Junction box		box	1	1.72	India Mart (https://www.indiamart.com/)

Table ST4: Material requirement and corresponding cost for module B

Component	Parts	Units	usage+ 10 wt % wastage	Material cost (USD/module)	Source
FTO Glass	conductivity > 7 per square	m^2	1	55.13	India Mart (https://www.indiamart.com/)
FTO cleaning	liquid soap	ml	11.00	0.15	Quote by Jaiswal chemicals
	DI water	ml	11.00	0.15	Quote by Jaiswal chemicals
	Ethanol	ml	11.00	0.38	Quote by Jaiswal chemicals
c-SnO_2	Blocking layer		0.22	11.86	Quote by Jaiswal chemicals
NiOx	NiOx paste	g	1.79	28.85	Solaronix
Carbon paste (blade coating)	ink	g	14.38	1.82	India Mart (https://www.indiamart.com/)
Perovskite	$Pb(NO_3)_2$	g	0.23	0.005	[1]
	KI	g	0.24	0.014	[1]
	C_3H_7NO	g	0.79	0.001	[1]
	CH_3NH_2	g	0.02	0.001	[1]
	HI	g	0.09	0.044	[1]
	$(CH_3)_2SO$	g	0.20	0.001	[1]
Solder wire	Sn	g	1.07	0.011	[1]
Edge seal	Al	g	1555	3.84	[1]
Lamination	EVA	g	138	1.51	[1]
Edge sealant	butyl rubber	g	10.5	0.037	[1]
Back glass	2mm glass	m^2	1	2.96	[1]
Junction box		box	1	1.72	India Mart (https://www.indiamart.com/)

References:

[1] Song Z, McElvany CL, Phillips AB, Celik I, Krantz PW, Watthage SC, et al. A technoeconomic analysis of perovskite solar module manufacturing with low-cost materials and techniques. Energy Environ Sci 2017;10:1297–305. https://doi.org/10.1039/C7EE00757D.
